# Reflex Asthma, Especially of Nasal Origin1This paper was part of a discussion on Asthma, before the Buffalo Pathological Society January 17, 1890. The other papers were not presented for publication—(Editor.)

**Published:** 1890-03

**Authors:** Frank Hamilton Potter

**Affiliations:** Buffalo, N. Y.; Lecturer on Diseases of the Nose and Throat, Niagara University


### REFLEX ASTHMA, ESPECIALLY OF NASAL ORIGIN.[1]

By FRANK HAMILTON POTTER, M. D., Buffalo, N. Y.

Lecturer on Diseases of the Nose and Throat, Niagara University.

ASTHMA is a symptom. All attempts to give it a distinct pathology have failed. It is associated or dependent upon so many conditions, that it is frequently very difficult, especially in its early stages, to determine upon what changes its peculiar phenomena depend. It should, therefore, be studied as pain and cough are studied. It indicates a morbid process somewhere in the economy, generally, perhaps, in the respiratory organs, but not necessarily there. If the theory of Weber is correct—and there is no other that seems to fulfil so many of the elements of the case—then any cause which will produce a paralysis of the vaso-motor nerves, governing the calibre of the blood-vessels of the bronchial mucous membrane, will develop an attack of asthma. Just why a particular irritation in a given case should lead to this chain of events, we do not know. We accept as a fact that an indiscretion in diet, for instance, will sometimes produce an asthmatic paroxysm, just as we accept the observation that an accumulation of wax in the ear may be the cause of a persistent cough.

1. This paper was part of a discussion on Asthma, before the Buffalo Pathological Society January 17, 1890. The other papers were not presented for publication—(EDITOR.)

Dr. Andrew H. Smith[1] has reported a case of asthma, in which a lobster salad caused a paroxysm with unvarying certainty. He says, also, that these cases are by no means rare, and that the term dyspeptic asthma is an entirely legitimate one. Leaving out of consideration the causes acting directly upon the lungs, and also those arising from cardiac disease, we find that, besides the stomach just referred to, the starting point of an asthmatic attack may be in the intestines, upon the skin, in the mouth, the nose, and naso-pharynx. There is some relation, also, between the uremic, lithemic, and gouty constitutions and asthma. Trousseau reports the case of a boy of five years with undoubted asthma, who, two years afterward, developed a typical gouty arthritis, during which the attacks of asthma entirely disappeared. We see, therefore, that the subject of reflex asthma is a very wide one—altogether too wide to come within the limits of this paper, and too wide also to be covered by my experience. This paper will be limited to that class of cases of which I can speak from some personal observation, viz: Asthma depending wholly or in great part upon disease in the nose and naso-pharynx. And while this subject will be presented as strongly as possible, I ask you to remember that the other factors in the case are never lost sight of. In the present state of our knowledge, it seems necessary to assume in every case of asthma—

1st. A general neurotic condition as originally, I believe, advocated by Salter.

2d. A pathological area somewhere in the economy, and

3d. Some exciting cause, possibly in the atmosphere, possibly in the food, but generally unknown.

With these general propositions constantly in mind, we can discuss the irritations arising from any special region, without adopting the mistaken view that asthma is always dependant upon disease of this region, or always complicated by it.

About the same time that Weber announced his theory of vasomotor paresis, Voltolini reported the cure of a case of asthma by the removal of a mucous polyp from the nasal passages. And he is considered, almost universally, to have been the first to demonstrate the relationship between the upper and lower air-passages in this disease. John N. Mackenzie[2], however, has shown that this is erroneous, and has pointed out from the writings of Aurelian, Zecchius (1650), Schneider, Floyer, (1726), Bree, (1811), Trousseau, Follin and Duplay, and Feber, (1869), that the association of nasal disease and asthma was known long before the time of Voltolini. And he states that Feber, referring to the frequent association of sneezing, migraine,

1. New York *Medical Journal*, January 19, 1889.

2. Transactions of the American Laryngological Association, 1886-1887.

hay-fever, and bronchial asthma, advanced the theory that these phenomena were the expression of a neurosis of the trigeminus nerve—a theory that has recently been resurrected in a modified form by Schadewaldt. Voltolini, however, was the first to cure a patient by removing the nasal disease, and thus he definitely fixed attention upon the morbid conditions of the nose as often being an element in the production of asthma. Since then, much has been written upon this question, especially by Hanisch, Porter, Daly, Todd, Spencer, John N. Mackenzie, Roe, Schäffer, Bresgen, Hack, Bosworth, and others. The evidence is so large, and has been so carefully presented, as to place the subject beyond controversy. Next to the bronchial tubes, the most important area conceived in the respiratory vaso-motor disturbances is found in the nasal passages, and it only remains to determine what conditions there act as predisposing and exciting causes of asthma, in what way they do this, and what treatment is necessary to remove them. We know that the nose becomes an easy prey to morbid processes. This is so from its genesis, from its situation, and from its peculiar physiology. From its genesis we have errors of formation, from its situation it is liable to trauma, and from the important part it plays in respiration, in phonation, and in olfaction, we have an organ highly developed and very sensitive. When disturbed, it affects directly and indirectly the rest of the organism, especially the respiratory tract—directly, through the continuity of its lining membrane, and by developing, when obstructed, the necessity of mouth breathing; indirectly, through the nervous system. The relation between the nasal mucous membrane and the nervous system, was clearly formulated by Dr. Jacobi, in a paper[1] upon the effects of nasal polypi in children. His conclusions were:

1st. The trigeminus, with all its branches, is subjected to direct or reflex irritation, arising from the inflamed condition of the nasal mucous membrane.

2d. The thickening of the mucous membrane, in the narrow nasal passages of the child, and especially the presence of a polypus, seriously interferes with respiration, and the result is the accumulation of carbonic acid gas in the brain, particularly about the respiratory center at the medulla oblongata.

3d. The lymphatic system of the nasal mucous membrane and that of the dura-mater and the arachnoid membrane are in intimate relation with each other, which is so close that they can be injected from either side.

According to Roe[2] there are two forms of nasal disease which may establish this relationship and provoke attacks of asthma:

1. New York Medical Journal, 1883, p. 376.
2. Journal of the American Medical Association, September 15, 1883.

1st. The most frequent form resulting from narrowing or occlusion of the nasal passages by hypertrophied tissue or nasal polypi.

2d. That induced by disease of the pituitary mucous membrane, unassociated with hypertrophy or polypi.

The first is both mechanical and reflex in its character, while the second is purely reflex.

Bosworth—whose contributions are the latest as well as the most positive we have upon the subject—includes, besides hypertrophies and neoplasms, the bony deformities and deflections, and diseases of the naso-pharynx, as conditions that may lead to the development of asthma. He says that he has never seen a case of asthma without an obstructive lesion of the upper air-passages. He accepts the theory of vaso-motor paralysis, and explains the connection between the mucous membrane of the nose and that of the bronchial tubes as follows[1]: "The most intricate, the most delicate, and most important part of the whole respiratory tract lies in the nose, in that mass of blood-vessels which we call the turbinated tissues, and which serve to supply the inspired air with moisture, by pouring out upon the surface of the mucous membrane a large amount of water—sixteen ounces in the course of the day—by which the inspired air becomes saturated with moisture, this function being necessarily regulated with an extreme degree of nicety of adjustment. This establishes, in what way or through what nerves or ganglia I do not discuss, but to my mind does unquestionally establish a most intimate connection between the two portions of the respiratory tract. The blood supply in the nose being regulated by the same vaso-motor tract as that which regulates the blood supply of the bronchial tubes, a disturbance in one region is liable to be followed by a disturbance in the other; a morbid condition in one region renders the other especially susceptible to diseased processes. This, briefly, is the history of the connection between the two parts. Hence, we see, therefore, how a diseased condition in the nasal cavity may predispose a neurotic patient under favorable atmospheric conditions, to an attack of asthma; the same line of reasoning, as will be noted, being followed here as in the case of hay-fever." In support of these views, he presents the analysis of eighty cases of asthma, in every one of which there was found some obstructive lesion of the upper air passages. These were removed with the result of curing forty-seven, improving twenty-six, two remained unimproved, and five unknown.

I believe you will agree with the suggestion that Dr. Bosworth goes too far. The proposition that an obstructive lesion in the upper air passages *may* be a predisposing or even an exciting cause of an

1. Diseases of the Nose and Throat, volume i., p. 238.

asthmatic paroxysm cannot be controverted; but that is vastly different from saying that almost all cases of asthma are dependent upon or complicated by such lesions. It would take too long, and, indeed, it is not necessary to repeat what each of the writers above mentioned have said upon this subject. In the main, we can say that while the prevailing opinion is conservative as to the number, it is positive in the belief that in a certain percentage of cases of asthma, the principal fault is to be found in the nose or naso-pharynx. This being so, we can claim without qualification that all cases of this nature should be properly examined as to the condition of those organs, because, unless so examined, a prominent source of mischief may be overlooked to the great injury of the patient.

One more question remains to be considered, viz., the occasional association of urticaria, asthma, and coryza. On this point I cannot do better than to direct attention to the words of Mackenzie. Referring to the fact that the relation of asthma to skin affections was familiar long before the days of Trousseau, he says: "Thus the illustrious Hoffmann mentions, as a fact of common experience, that asthma sometimes follows the suppression of a cutaneous rash; and before him Baglini had recommended, in such an event, that the patient should sleep with one having the "scabies," that, catching it, he might be relieved of his asthma. It is also related that William of Orange was cured of an inveterate asthma during the running of a sore on the shoulder, produced by the famous cannon-ball wound received at the battle of the Boyne. In the condition of affairs we are discussing, the coryza may precede the asthma and urticaria in time of appearance, disappearing or remaining after their eruption; or the asthma or urticaria may antedate the attack of coryza; or, finally, instead of alternating the one with the other, they may appear simultaneously in the individual. These phenomena seemingly depend on an imperfectly defined neurosis or vaso-motor influence (possibly some derangement of the cervico-occipital sympathetic), which is probably the connecting link between these affections. Now, in attempting to define the reciprocal relationship between this triad of conditions, we may regard the skin essentially as a part of the respiratory tract,—as the external organ of respiration. It is only necessary for me to recall the physiological importance of the skin in respiration among some of the lower animals, and the embarrassment of respiration in man from pathological or experimental suppression of the cutaneous function. We may accordingly regard this neuro-vascular disturbance of the external surface as a natural symptom of the respirator, vaso-motor neuroses, and

1. Op. cit.

assume that, while the relation of asthma and coryza may be explicable by a possible normal sympathy existing between the two extremities of the internal respiratory tract, both asthma and coryza may be linked to the skin affection by a sympathetic bond which holds in equilibrium and close consent the whole mechanism of the respiratory function."

In conclusion, the treatment of the lesions, under discussion, demands a few words. We have seen that they are essentially obstructive, and are generally found in the nose and naso-pharynx. They must be removed by surgical means. Medical measures are at the best unsatisfactory and tedious—generally they fail entirely. It is not necessary to consider the method of the operations now usually employed to remove obstructive lesions in these parts, but simply to say that according as the latter are of the hard or soft tissues, we use the saw, trephine, and chisel, or the snare, scissors, and cautery. These measures have been, and still are, severely criticised in some quarters. As an example, the words of Lemoyez[1] may be quoted: "The nose is becoming everything, is encroaching upon, and threatening to swallow up all pathology. Before yesterday asthma, yesterday stridulous laryngitis, to-day aprosexia are made tributary to it. In the name of progress, everything is snared or cut away." This is witty, but it is not true. In so far as it is proposed to treat in a surgical manner the diseases of the nose that may be causative factors in the production of asthma, there is no thought of proceeding upon any new or strange surgical principles. Certainly, no one would think of attempting to correct deformities of the bones, or remove neoplasms, or restore obstructive channels elsewhere by the use of powders, sprays, washes, and salves; neither can these things accomplish much in removing the same lesions when found in the nose. If you have a case of asthma dependent upon or complicated by an obstructed nostril with pressure between the middle turbinated bone and the septum, for instance, you must operate before you can expect to remove that element from the case. I am not defending nasal surgery in general; that is another question, but specially with reference to the subject in hand. It has been proved that some cases of asthma result from obstructions in the upper air passages. These obstructions are of many kinds, and act in a peculiar and complicated manner. It is, therefore, necessary to ascertain in all cases of asthma, whether they are present or not. If they are, I believe that if properly removed, many cases of this disease would yield to treatment, and the percentage of recoveries be much larger than it is to-day.

---

1. The Journal of Laryngology, June, 1888, p. 231.

